# 8-Aminoinosine and 8-Aminohypoxanthine Inhibit Purine Nucleoside Phosphorylase and Exert Diuretic and Natriuretic Activity

**DOI:** 10.1124/jpet.122.001221

**Published:** 2022-08

**Authors:** Edwin K. Jackson, Elizabeth V. Menshikova, Vladimir B. Ritov, Zaichuan Mi, Lori A. Birder

**Affiliations:** Department of Pharmacology and Chemical Biology (E.K.J., E.V.M., V.B.R., Z.M.) and Department of Medicine (L.A.B.), University of Pittsburgh School of Medicine, Pittsburgh, Pennsylvania

## Abstract

8-Aminoguanine and 8-aminoguanosine (via metabolism to 8-aminoguanine) are endogenous 8-aminopurines that induce diuresis, natriuresis, and glucosuria by inhibiting purine nucleoside phosphorylase (PNPase); moreover, both 8-aminopurines cause antikaliuresis by other mechanisms. Because 8-aminoinosine and 8-aminohypoxanthine are structurally similar to 8-aminoguanosine and 8-aminoguanine, respectively, we sought to define their renal excretory effects. First, we compared the ability of 8-aminoguanine, 8-aminohypoxanthine, and 8-aminoinosine to inhibit recombinant PNPase. These compounds inhibited PNPase with a potency order of 8-aminoguanine > 8-aminohypoxanthine = 8-aminoinosine. Additional studies showed that 8-aminoinosine is a competitive substrate that is metabolized to a competitive PNPase inhibitor, namely 8-aminohypoxanthine. Administration of each 8-aminopurine (33.5 µmol/kg) reduced the guanine-to-guanosine and hypoxanthine-to-inosine ratios in urine, a finding confirming their ability to inhibit PNPase in vivo. All three 8-aminopurines induced diuresis, natriuresis, and glucosuria; however, the glucosuric effects of 8-aminohypoxanthine and 8-aminoinosine were less pronounced than those of 8-aminoguanine. Neither 8-aminohypoxanthine nor 8-aminoinosine altered potassium excretion, whereas 8-aminoguanine caused antikaliuresis. In vivo administration of 8-aminoinosine increased 8-aminohypoxanthine excretion, indicating that 8-aminohypoxanthine mediates, in part, the effects of 8-aminoinosine. Finally, 8-aminohypoxanthine was metabolized to 8-aminoxanthine by xanthine oxidase. Using ultraperformance liquid chromatography–tandem mass spectrometry, we identified 8-aminoinosine as an endogenous 8-aminopurine. In conclusion, 8-aminopurines have useful pharmacological profiles. To induce diuresis, natriuresis, glucosuria, and antikaliuresis, 8-aminoguanine (or its prodrug 8-aminoguanosine) would be preferred. If only diuresis and natriuresis, without marked glucosuria or antikaliuresis, is desired, 8-aminohypoxanthine or 8-aminoinosine might be useful. Finally, here we report the in vivo existence of another pharmacologically active 8-aminopurine, namely 8-aminoinosine.

**SIGNIFICANCE STATEMENT:**

Here, we report that a family of 8-aminopurines affects renal excretory function: effects that may be useful for treating multiple diseases including hypertension, heart failure, and chronic kidney disease. For diuresis and natriuresis accompanied by glucosuria and antikaliuresis, 8-aminoguanine (or its prodrug 8-aminoguanosine) would be useful; if only diuresis and natriuresis is called for, 8-aminohypoxanthine or 8-aminoinosine would be useful. Previously, we identified 8-aminoguanine and 8-aminoguanosine as endogenous 8-aminopurines; here, we extend the family of endogenous 8-aminopurines to include 8-aminoinosine.

## Graphical abstract

**Figure:**
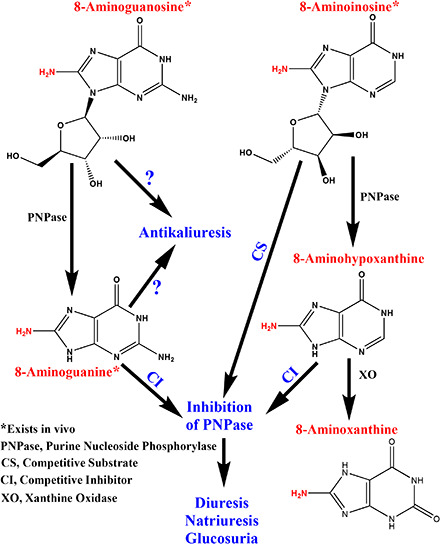

## Introduction

Studies have established the in vivo existence of a family of 8-nitro and 8-amino substituted guanosines and guanines. In this regard, investigators have reported the presence of 8-nitroguanosine (Akaike et al., 2003) and 8-aminoguanosine (Sodum et al., 1993) in tissues and the existence of 8-hydroxyguanosine (Park et al., 1992), 8-nitroguanine (Ohshima et al., 2006), 8-hydroxyguanine (Fraga et al., 1990), and 8-hydroxy-2’-deoxyguanosine (Lam et al., 2012) in urine. Recently, we detected, using ultraperformance liquid chromatography–tandem mass spectrometry (UPLC-MS/MS), both 8-aminoguanosine and 8-aminoguanine in urine and in the renal cortical and medullary interstitium (Jackson and Mi, 2017).

Because of the lack of knowledge regarding the pharmacological effects of endogenous 8-hydroxy, 8-nitro, and 8-amino substituted guanine-based purines on the cardiovascular system and kidneys, in 2016 we systematically assessed in rats the effects of an array of these compounds on the cardiovascular system and kidneys (Jackson et al., 2016). We observed that of all the compounds tested, 8-aminoguanosine and 8-aminoguanine were the most pharmacologically active. Both compounds induced diuresis, increased the excretion of sodium and glucose, decreased the excretion of potassium, and attenuated deoxycorticosterone/salt hypertension (Jackson et al., 2016). In subsequent studies, we also discovered that chronic treatment with 8-aminoguanine reverses age-associated pathology of the lower urinary tract (i.e., reverses age-associated pathologic changes in the histology, ultrastructure, and function of the bladder) (Birder et al., 2020). We have also found that 8-aminoguanosine/guanine improves pulmonary hypertension and sickling of red blood cells in blood from patients living with sickle cell anemia (Jackson and Tofovic, 2020).

Our work to date supports our hypothesis that most of the effects of 8-aminoguanosine, except for its antikaliuretic actions, are mediated via its conversion to 8-aminoguanine (i.e., 8-aminoguanosine is a prodrug) (Jackson and Mi, 2017). Also, our findings to date suggest that most of the effects of 8-aminoguanine are mediated by inhibition of purine nucleoside phosphorylase (PNPase), with the exception of antikaliuresis, which may in part be mediated by inhibition of Rac1 signaling (Jackson et al., 2018).

Inosine differs from guanosine only in the lack of an amino group in the two position of the purine ring, and likewise, hypoxanthine (metabolite of inosine) differs from guanine (metabolite of guanosine) in the same manner. Thus, the structural parallel between the 8-aminoguanosine/8-aminoguanine pair and the 8-aminoinosine/8-aminohypoxanthine pair is striking and suggests that 8-aminoinosine and 8-aminohypoxanthine may be pharmacologically active. Here, we considered the hypothesis that the family of pharmacologically active 8-aminopurines may include 8-aminoinosine and 8-aminohypoxanthine.

## Methods

### Materials

8-Aminoguanine was from Toronto Research Chemicals (Toronto, Ontario, Canada); 8-aminoinosine and 8-aminohypoxanthine were from Santa Cruz Biotechnology, Inc. (Dallas, TX); recombinant human purine nucleoside phosphorylase (rhPNPase) was from R&D Systems (Minneapolis, MN); xanthine oxidase, inosine, hypoxanthine, guanosine, guanine, and uric acid were from MilliporeSigma (St. Louis, MO); ^15^N_4_-inosine and ^13^C_5_-hypoxanthine were from Cambridge Isotope Laboratories, Inc. (Tewksbury, MA); and ^13^C_10_,^15^N_5_-guanosine; ^13^C_2_,^15^N-guanine were from Medical Isotopes, Inc. (Pelham, NH).

### Animals

Animal experiments used male Sprague-Dawley rats from Charles River (Wilmington, MA) that were approximately 16 weeks old. The Institutional Animal Care and Use Committee approved all procedures. The investigation conforms to National Institutes of Health Guide for the Care and Use of Laboratory Animals.

### Assessment of Inhibitory Effects of 8-Aminopurines on the Activity of rhPNPase

rhPNPase was incubated for 10 minutes at 30°C in 50 µl of medium containing 100–2000 µmol/L of inosine or guanosine, 0.1 mg/ml of bovine serum albumin, and 50 mmol/L of KH_2_PO_4_ (pH 7.4) in the absence or presence of 8-aminoguanine (25 or 50 µmol/L), 8-aminohypoxanthine (25, 50, or 100 µmol/L), or 8-aminoinosine (25, 50 or 100 µmol/L). The concentration of PNPase used in these reactions was very low, i.e., 1 ng per 50 µl, to minimize substrate depletion and product production and to ensure that initial reaction velocities were linear over the reaction time. Reactions were rapidly terminated by placing the sample tubes in a 90°C water bath. Products (hypoxanthine or guanine) were analyzed by high pressure liquid chromatography (HPLC) with UV absorbance, and results were fitted to a competitive inhibition model using GraphPad Prism 9 for Windows (San Diego, CA).

### Michaelis-Menten Kinetics for Metabolism of Inosine and 8-Aminoinosine by rhPNPase

rhPNPase (1 ng) was incubated for 10 minutes at 30°C in 50 µl of medium containing 100–2000 µmol/L of inosine or 8-aminoinosine, 0.1 mg/ml of bovine serum albumin, and 50 mmol/L of KH_2_PO_4_ (pH 7.4). Reactions were terminated by rapidly placing the sample tubes in a 90°C water bath. Products (hypoxanthine or 8-aminohypoxanthine) were analyzed by HPLC with UV absorbance. To determine Michaelis constant (Km) and maximum reaction velocity (Vmax), results were fitted to the Michaelis-Menten equation using GraphPad Prism 9 for Windows.

### Inhibition of rhPNPase-Induced Conversion of 8-Aminoinosine to 8-Aminohypoxanthine by Inosine

rhPNPase (5 ng) was incubated for 10 minutes at 30°C in 50 µl of medium containing 200–2000 µmol/L of inosine, 0.1 mg/ml of bovine serum albumin, and 50 mmol/L of KH_2_PO_4_ (pH 7.4) in the presence of 25, 50, or 100 µmol/L of 8-aminoinosine. 8-Aminoinosine and 8-aminohypoxanthine were analyzed by HPLC with UV absorbance.

### Metabolism of 8-Aminohypoxanthine versus Hypoxanthine by Xanthine Oxidase

Xanthine oxidase (50 ng) was incubated for 10 minutes at 30°C in 50 µl of medium containing 60–2000 µmol/L of hypoxanthine or 8-aminohypoxanthine, 0.1 mg/ml of bovine serum albumin, and 50 µmol/L of KH_2_PO_4_ (pH 7.4). Xanthine, uric acid, and 8-aminoxanthine were analyzed by HPLC with UV absorbance, and the rate of product formation was determined (xanthine plus uric acid for hypoxanthine as substrate and 8-aminoxanthine for 8-aminohypoxanthine as substrate). To determine Km and Vmax, results were fitted to the Michaelis-Menten equation using GraphPad Prism 9 for Windows (San Diego, CA).

### Analysis of Purines by High Pressure Liquid Chromatography with UV Absorbance

Hypoxanthine, xanthine, 8-aminoxanthine, 8-aminohypoxanthine, 8-aminoinosine, and guanine were measured by HPLC analysis using an Agilent (Santa Clara, CA) HPLC system, which included a HP Agilent Technologies 1100 series HPLC chromatograph equipped with a diode array detector (model G1315B) and autosampler (model G1313A ALS). Samples were heated to 90°C for 1.5 minutes (to inactivate enzymes), vortexed, and centrifuged at 14,000 rpm for 25 minutes at 4°C, and the supernatants were collected and transferred into HPLC vials. Aliquots of samples (2–15 μl) were injected onto a C-18 reverse phase column (Agilent Eclipse Plus C18, 5 μm, 4.6 × 250 mm), which was protected by a guard cartridge. Analysis was conducted in gradient mode [buffer A: 0.15 M KH_2_PO_4_ in water (pH 6.0); buffer B: 0.15 M KH_2_PO_4_ in 15% acetonitrile (pH 6.0); and linear gradient (percent B): at 0 minutes, 3.0%; from 0 to 9 minutes, 3.0%; from 9 to 25 minutes, to 100.0%; from 25 to 30 minutes, 100.0%; from 30 to 31 minutes, to 3.0%; and from 31 to 40 minutes, 3.0%]. The flow rate was 0.8 ml/min. Purines in the eluate were monitored at 259 nm. Chromatograms were processed and stored in digital form with Agilent OpenLAB CDS software. Standard curves were generated from authentic standards.

### UPLC-MS/MS Assay

Purines in urine were measured by UPLC-MS/MS as previously described (Jackson et al., 2009), with modifications. Urine samples were diluted 1:30 in pure water and heavy-isotope internal standards were added (^15^N_4_-inosine, ^13^C_5_-hypoxanthine, ^13^C_10_,^15^N_5_-guanosine, and ^13^C_2_,^15^N-guanine). Samples were injected into a Waters UPLC BEH C18 column (1.7 µm beads; 2.1 × 150 mm; Milford, MA), and purines were separated with a mobile phase (flow rate of 300 uL/min) consisting of a linear gradient of 1% acetic acid in water (pH, 3; mobile phase A) and 100% methanol (mobile phase B). The mobile phase was delivered with a Waters Acquity UPLC system with gradient (A/B) settings as follows: from 0 to 2 minutes, 99.6%/0.4%; from 2 to 3 minutes, to 98.0%/2.0%; from 3 to 4 minutes, to 85.0%/15.0%; and from 4 to 6.5 minutes, to 99.6%/0.4%. Samples were quantified by multiple reaction monitoring with a TSQ Quantum-Ultra triple quadrupole mass spectrometer (ThermoFisher Scientific, San Jose, CA) with an electrospray ionization source. The instrument parameters were: sample tray temperature, 10°C; column temperature, 50°C; ion spray voltage, 4.0 kilovolts; ion transfer tube temperature, 350°C; source vaporization temperature, 320°C; Q2 CID gas, argon at 1.5 mTorr; sheath gas, nitrogen at 60 psi; auxillary gas, nitrogen at 35 psi; Q1/Q3 width, 0.7/0.7 units full-width half-maximum; scan width, 0.6 units; and scan time, 0.01 seconds. The internal standards for guanosine, guanine, inosine, and hypoxanthine were ^13^C_10_,^15^N_5_-guanosine, ^13^C_2_,^15^N-guanine, ^15^N_4_-inosine, and^13^C_5_-hypoxanthine, respectively. Also, ^15^N_4_-inosine and^13^C_5_-hypoxanthine were used as internal standards for 8-aminoinosine and 8-aminohypoxanthine, respectively. Quantification was achieved via interpolation from standard curves.

### Animal Protocol

Rats were anesthetized with Inactin (90 mg/kg intraperitoneally), placed on an isothermal pad, and their temperature was monitored with a rectal probe thermometer. Body temperature was kept at approximately 37°C by adjusting the distance of a heat lamp from the surgical preparation. To maintain a clear airway, a polyethylene (PE)-240 cannula was placed in the trachea. To monitor mean arterial blood pressure (MABP) and heart rate (HR), the carotid artery was cannulated with PE-50 tubing, which was connected to a digital blood pressure analyzer (Micro-Med, Inc., Louisville, KY). For intravenous access, a PE-50 cannula was inserted into the jugular vein, and to maintain hemodynamic stability, an infusion of 0.9% saline (25 μl/min) was initiated. For timed urine collections, the left ureter was cannulated with PE-10 tubing. For measurement of renal blood flow (RBF) and mesenteric blood flow (MBF), transit-time flow probes (Transonic Systems, Inc., Ithaca, NY) were placed around the renal artery (1 mm probe) and mesenteric artery (2 mm probe), and the cables from these probes were connected to a T-206 model Transonic Systems flowmeter. After instrumentation, the animal was allowed a rest period of approximately 1 hour. The experiment involved three experimental periods, during which time urine was collected for 30 minutes, and MABP, HR, RBF, and MBF were time averaged. Period 1 was from 0 to 30 minutes into the protocol, period 2 was from 40 to 70 minutes into the protocol, and period 3 was from 85 to 115 minutes into the protocol. Immediately after period 1, animals received an intravenous injection of either vehicle (vehicle/time control), 8-aminoguanosine (33.5 µmoles/kg; 1 ml/kg), 8-aminohypoxanthine (33.5 µmoles/kg; 1 ml/kg), or 8-aminoinosine (33.5 µmoles/kg; 1 ml/kg). The dose of 33.5 µmoles/kg was chosen based on our previously published findings that this dose of 8-aminoguanosine and 8-aminoguanine induced robust changes in renal excretory function with little acute effects on systemic hemodynamics (Jackson et al., 2016). Rats were randomly assigned to one of the four treatment groups, and each rat received only a single treatment. Urine levels of sodium and potassium were measured using flame photometry (Model IL-943; Instrumentations Laboratory Inc., Lexington, MA), and urine levels of glucose were assessed using a colorimetric assay (Glucose Colorimetric Assay Kit; catalog number 10009582; Cayman Chemical).

### Statistics

Statistical analysis was performed using one-factor ANOVA, with either independent sampling or repeated measures as appropriate, followed by a Bonferroni test if effects in the ANOVA were significant. One rat in the vehicle group had a baseline sodium excretion that was approximately 10-fold greater than the baseline values for other rats; therefore, a replacement experiment was performed for this group. The statistical outcomes for both the original vehicle group and the group with the replacement experiment were the same. Statistical comparisons of Km and Vmax using different substrates were performed using GraphPad Prism 9 for Windows to determine whether the nonlinear regression best-fit value of a parameter differed between substrates. The criterion of significance was *P* < 0.05. Values are presented as means and S.E.M.

## Results

### Inhibitory Effects of 8-Aminopurines on rhPNPase

**Fig. 1.**
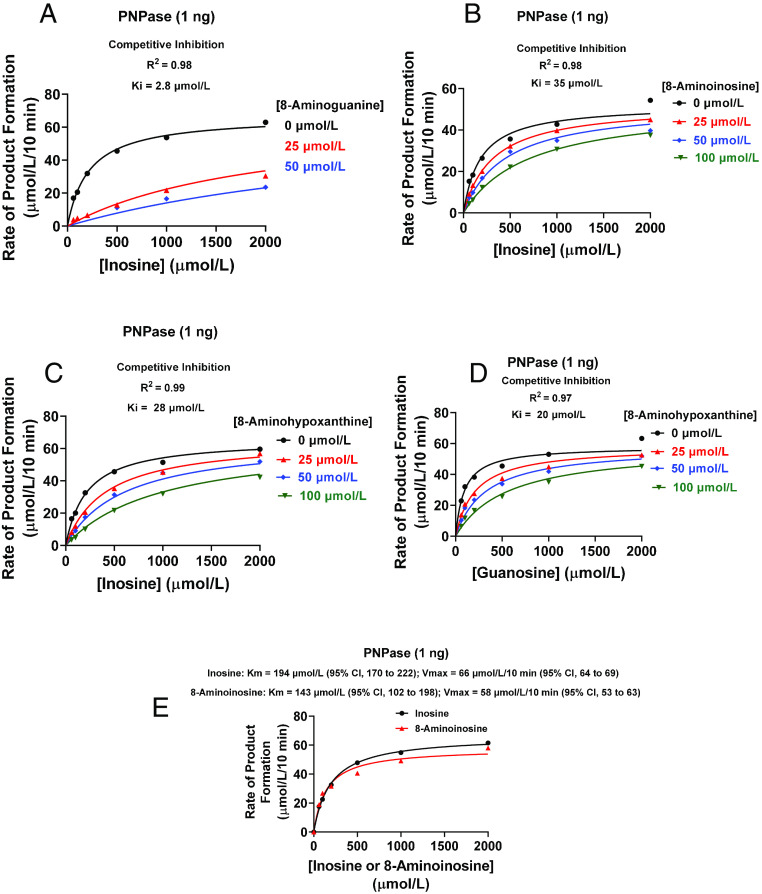
Effects of 8-aminoguanine, 8-aminohypoxanthine, and 8-aminoinosine on the activity of rhPNPase. rhPNPase (1 ng) was incubated for 10 minutes at 30°C in 50 µl of medium containing 100–2000 µmol/L of inosine or guanosine, 0.1 mg/ml of bovine serum albumin, and 50 mmol/L of KH_2_PO_4_ (pH 7.4) in the presence of the indicated concentrations of (A) 8-aminoguanine, (B) 8-aminoinosine, or (C and D) 8-aminohypoxanthine. Product (hypoxanthine for inosine as substrate and guanine for guanosine as substrate) was analyzed by HPLC with UV absorbance, and results were fitted to a competitive inhibition model using GraphPad Prism 9 for Windows (San Diego, CA). (E) rhPNPase (1 ng) was incubated for 10 minutes at 30°C in 50 µl of medium containing 100–2000 µmol/L of inosine or 8-aminoinosine, 0.1 mg/ml of bovine serum albumin, and 50 mmol/L of KH_2_PO_4_ (pH 7.4). Hypoxanthine and 8-aminohypoxanthine were analyzed by HPLC with UV absorbance. To determine Km and Vmax, results were fitted to the Michaelis-Menten equation using GraphPad Prism 9 for Windows (San Diego, CA). The kinetic properties (Km and Vmax) of rhPNPase for inosine were similar to those for hypoxanthine (*n* = 3 repetitions; S.E.M. within symbol width), thus suggesting that 8-aminoinosine is a competitive substrate.

Here, we report the results of the first head-to-head comparison (i.e., under identical conditions) of 8-aminoguanine, 8-aminoinosine, and 8-aminohypoxanthine as competitive antagonists of PNPase, with inosine or guanosine as substrate. As illustrated in Fig. 1, all three 8-aminopurines exhibited competitive inhibition kinetics against the metabolism of inosine to hypoxanthine by rhPNPase. With inosine as substrate, the calculated inhibition constant (Ki) for 8-aminoguanine was 2.8 µmol/L [95% confidence interval (CI), 2.2–3.5; Fig. 1A], a value similar to that reported by Chern et al. (1993). While this work was in progress, Rabuffetti et al. (2021) reported that 8-aminoinosine is a competitive inhibitor of rhPNPase, with a Ki of 48 µmol/L. Indeed, we also observed that 8-aminoinosine reduced the activity of rhPNPase (with inosine as substrate) in a manner consistent with competitive inhibition (Fig. 1B) and with a Ki of 35 µmol/L (95% CI, 26–49). Stoeckler et al. (1982) examined the inhibitory effects of 8-aminohypoxanthine on PNPase isolated from red blood cells and reported a Ki of 10 µmol/L. Using rhPNPase, we obtained a Ki for 8-aminohypoxanthine of 28 µmol/L (95% CI, 23–34; Fig. 1C) with inosine as substrate and 20 µmol/L (95% CI, 14–29; Fig. 1D) with guanosine as substrate. Thus, when tested under identical conditions using a high-quality recombinant PNPase, 8-aminoinosine and 8-aminohypoxanthine were potent antagonists of PNPase yet were approximately 10-fold less potent than 8-aminoguanine.

**Fig. 2.**
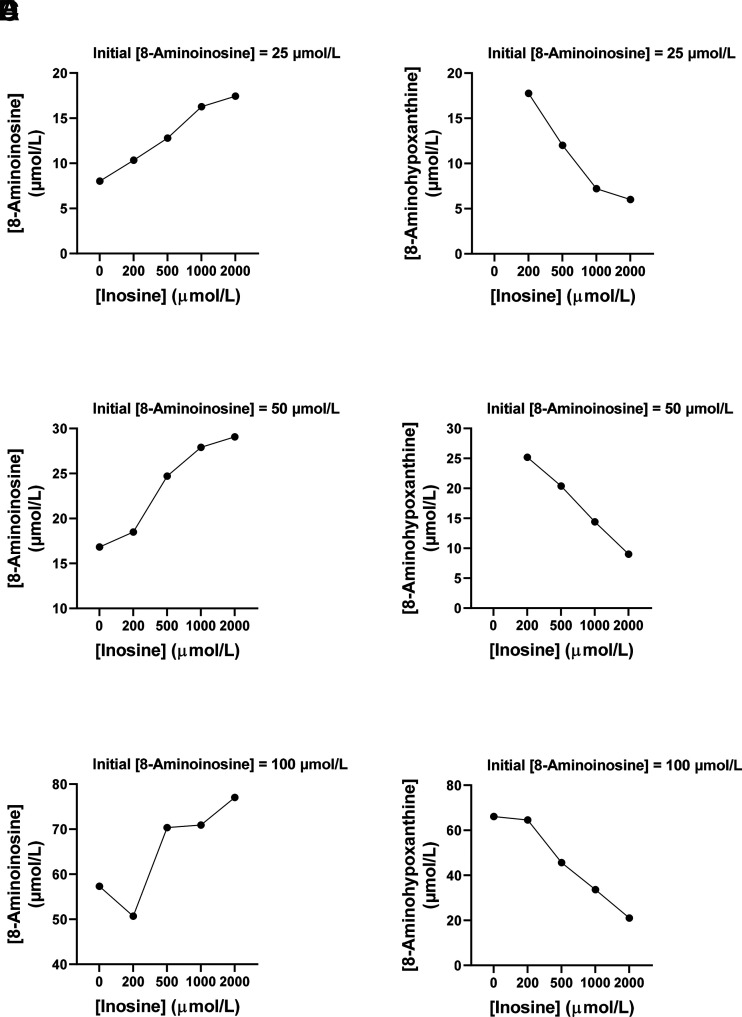
Inosine inhibits the metabolism of 8-aminoinosine by rhPNPase. rhPNPase (5 ng) was incubated for 10 minutes at 30°C in 50 *µ*l of medium containing 0–2000 µmol/L of inosine, 0.1 mg/ml of bovine serum albumin, and 50 mmol/L of KH_2_PO_4_ (pH 7.4) in the presence of (A and B) 25, (C and D) 50, or (E and F) 100 µmol/L of 8-aminoinosine. 8-Aminoinosine and 8-aminohypoxanthine were analyzed by HPLC with UV absorbance. After 10 minutes of incubation of 8-aminoinosine with rhPNPase, concentrations of 8-aminoinosine were much lower (A, C, and E) than the initial added concentrations and much (B, D, and F) of the initially added 8-aminoinosine was converted to 8-aminohypoxanthine. The addition of increasing concentrations of inosine (A, C, and E) inhibited the loss of 8-aminoinosine and (B, D, and F) reduced the formation of 8-aminohypoxanthine, indicating competition between inosine and 8-aminoinosine as substrates for rhPNPase.

We considered the hypothesis that 8-aminoinosine is a competitive substrate, rather than a true competitive inhibitor, of rhPNPase. This hypothesis was tested using two approaches. First, we performed a Michaelis-Menten kinetic analysis of rhPNPase with inosine as substrate and hypoxanthine as product versus 8-aminoinosine as substrate and 8-aminohypoxanthine as product. This analysis showed that the Km of rhPNPase with 8-aminoinosine as substrate was similar (overlapping 95% CIs) to the Km with inosine as substrate (Fig. 1E). Also, a statistical comparison of the best-fit Km for inosine versus the best-fit Km for 8-aminoinosine showed no significant difference. The Vmax of rhPNPase with 8-aminoinosine as substrate (58 µmol/L per 10 minutes; 95% CI, 53–63) was only slightly, but significantly, less than the Vmax with inosine as substrate (66 µmol/L per 10 minutes; 95% CI, 64–69). Second, we reasoned that if 8-aminoinosine and inosine are competitive substrates, the metabolism of 8-aminoinosine to 8-aminohypoxanthine by rhPNPase should be reversed by inosine in a concentration-dependent manner. To test this, we incubated for 10 minutes 8-aminoinosine (25, 50, or 100 µmol/L) with rhPNPase (5 ng per 50 µl) without and with various concentrations of inosine (200–2000 µmol/L). In the absence of inosine and at the end of the incubation, 8-aminoinosine concentrations were only a fraction of the initially added concentrations of 25, 50, and 100 µmol/L (Fig. 2, A, C, and E, respectively). Also, much of the initially added 8-aminoinosine (25, 50 and 100 µmol/L) was recovered as 8-aminohypoxanthine (Fig. 2, B, D, and F, respectively). Addition of increasing concentrations of inosine to the reaction inhibited the loss of 8-aminoinosine (Fig. 2, A, C, and E) and reduced the formation of 8-aminohypoxanthine (Fig. 2, B, D, and F). These data indicated that inosine competitively inhibits the metabolism of 8-aminoinosine to 8-aminohypoxanthine and establishes 8-aminoinosine as a competitive substrate of PNPase. Moreover, the fact that 8-aminohypoxanthine is a competitive antagonist of PNPase indicates that 8-aminoinosine can block PNPase activity via two mechanisms: 1) as a competitive substrate and 2) via conversion to 8-aminohypoxanthine, a potent competitive antagonist.

### Effects of 8-Aminopurines on the Guanine-to-Guanosine and Hypoxanthine-to-Inosine Ratios in Urine

**Fig. 3.**
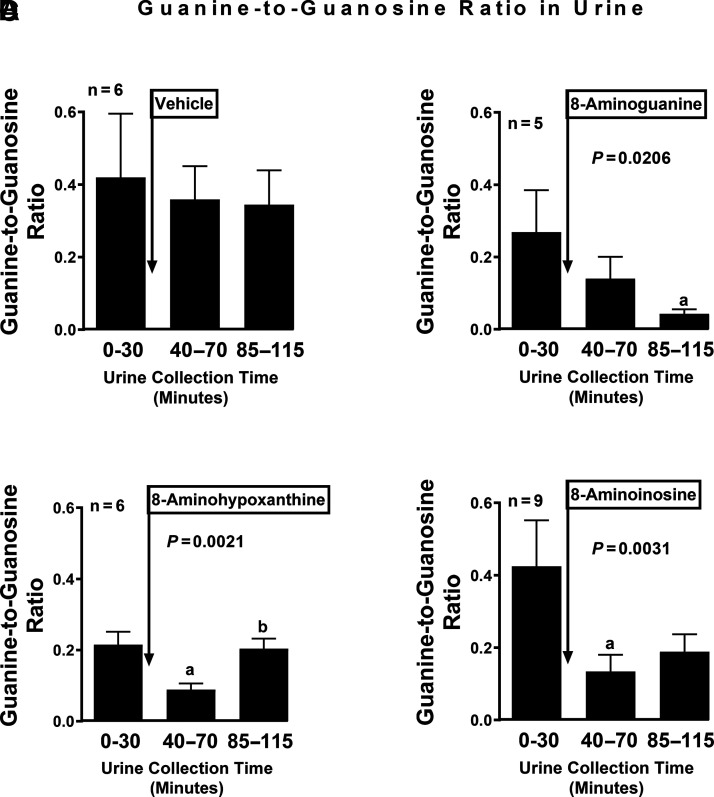
Effects of 8-aminoguanine, 8-aminohypoxanthine, and 8-aminoinosine on the guanine-to-guanosine ratio in urine. After a 30-minute basal period, intravenous boluses of (A) vehicle (*n* = 6), (B) 8-aminoguanine (33.5 µmol/kg, *n* = 5), (C) 8-aminohypoxanthine (33.5 µmol/kg, *n* = 9), or (D) 8-aminoinosine (33.5 µmol/kg, *n* = 6) were administered to anesthetized rats. Urine was collected at the indicated time intervals and analyzed for guanine and guanosine levels. The guanine-to-guanosine ratio was calculated by dividing the concentration of guanine in the urine by the concentration of guanosine in the urine. Values are means and S.E.M. for the indicated samples size (n). *P* values in panels are from repeated-measures one-factor ANOVA. Bonferroni tests: a, *P* < 0.05 versus 0–30 minutes; b, *P* < 0.05 versus 40–70 minutes.

**Fig. 4.**
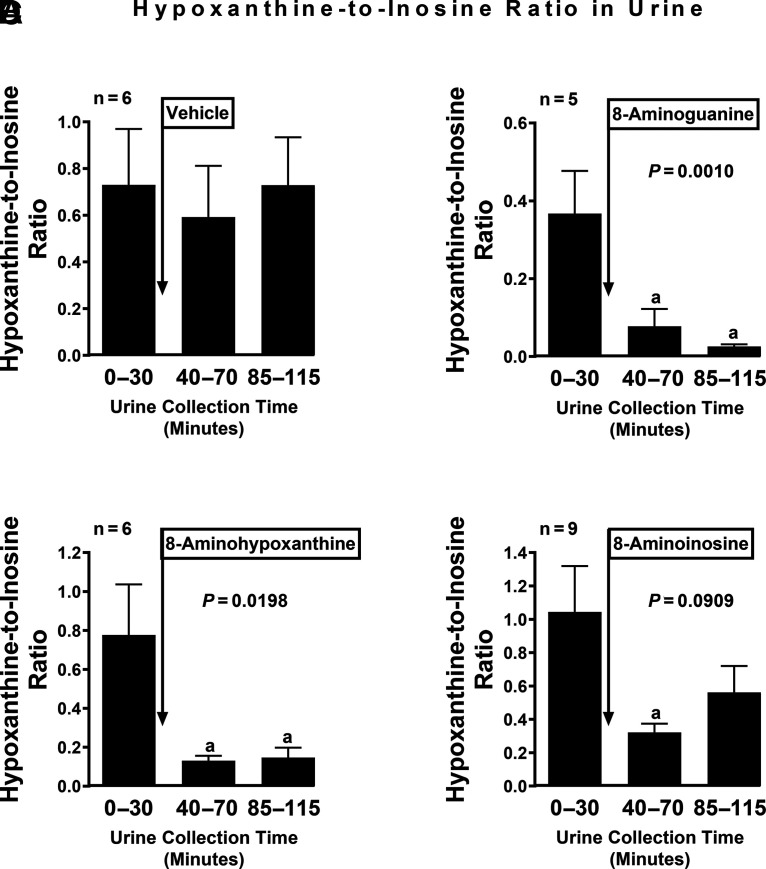
Effects of 8-aminoguanine, 8-aminohypoxanthine, and 8-aminoinosine on the hypoxanthine-to-inosine ratio in urine. After a 30-minute basal period, intravenous boluses of (A) vehicle (*n* = 6), (B) 8-aminoguanine (33.5 µmol/kg, *n* = 5), (C) 8-aminohypoxanthine (33.5 µmol/kg, *n* = 9), or (D) 8-aminoinosine (33.5 µmol/kg, *n* = 6) were administered to anesthetized rats. Urine was collected at the indicated time intervals and analyzed for hypoxanthine and inosine levels. The hypoxanthine-to-inosine ratio was calculated by dividing the concentration of hypoxanthine in the urine by the concentration of inosine in the urine. Values are means and S.E.M. for the indicated samples size (n). *P* values in panels are from repeated-measures one-factor ANOVA. Bonferroni tests: a, *P* < 0.05 versus 0–30 minutes.

PNPase metabolizes guanosine to guanine and inosine to hypoxanthine. Therefore, to assess whether systemic administration of 8-aminohypoxanthine or 8-aminoinosine inhibits PNPase activity in vivo, we measured using UPLC-MS/MS the ratio of PNPase products (guanine and hypoxanthine) to PNPase substrates (guanosine and inosine, respectively). For comparison, we included an additional group of animals treated with 8-aminoguanine, a well established PNPase inhibitor (Chern et al., 1993), and to rule out time or vehicle effects, we included a group of animals treated only with the vehicle for these 8-aminopurines. Vehicle per se did not alter the guanine-to-guanosine (Fig. 3A) or the hypoxanthine-to-inosine (Fig. 4A) ratios. In contrast, intravenous administration 8-aminoguanine, 8-aminohypoxanthine, and 8-aminoinosine (each at 33.5 µmoles/kg) suppressed the guanine-to-guanosine ratio (Fig. 3, B, C, and D, respectively), as well as the hypoxanthine-to-inosine ratio (Fig. 4, B, C, and D, respectively). Consistent with the enzyme inhibition kinetic analysis, the suppression of the guanine-to-guanosine ratio was more profound during the third urine collection period (85–115 minutes into the protocol) for 8-aminoguanine compared with 8-aminohypoxanthine or 8-aminoinosine (guanine-to-guanosine ratios during the third period for 8-aminoguanine, 8-aminohypoxanthine, and 8-aminoinosine were 0.04 ± 0.01, 0.20 ± 0.03, and 0.19 ± 0.05, respectively; one-factor ANOVA, *P* = 0.0055; Bonferroni test, *P* < 0.05 for 8-aminoguanine compared with either 8-aminohypoxanthine or 8-aminoinosine). Similarly, the suppression of the hypoxanthine-to-inosine ratio was more profound during the third urine collection period (85–115 minutes into the protocol) for 8-aminoguanine compared with 8-aminohypoxanthine or 8-aminoinosine (hypoxanthine-to-inosine ratios during the third period for 8-aminoguanine, 8-aminohypoxanthine, and 8-aminoinosine were 0.03 ± 0.01, 0.15 ± 0.05, and 0.56 ± 0.16, respectively; one-factor ANOVA, *P* < 0.0001; Bonferroni test, *P* < 0.05 for 8-aminoguanine compared with either 8-aminohypoxanthine or 8-aminoinosine).

### Effects of 8-Aminopurines on Urine Volume

Administration of the vehicle for 8-aminopurines did not alter the excretion rate of urine (Table 1), which indicated the stability of our experimental preparation. 8-Aminoguanine, 8-aminoinosine, and 8-aminohypoxanthine significantly (*P* = 0.0003, *P* = 0.0012, *P* < 0.0001, respectively) increased urine volume (Table 1). These results demonstrate that all three 8-aminopurines are diuretics.

**TABLE 1:** Effects of 8-aminopurines on urine volume (all values are in units of µl per 30 min) All groups received their respective treatments just after period 1 (basal period). The 8-aminopurines were administered intravenously at 33.5 µmol/kg. *P* values are from repeated-measures one-factor analysis of variance. Values are means ± S.E.M.

Treatment Group	Period 1 (0–30 min)	Period 2 (40–70 min)	Period 3 (85–115 min)	*P* Value
Vehicle group (n = 6)	94 ± 25	110 ± 19	119 ± 19	NS
8-Aminoguanine (n = 5)	75 ± 24	48 ± 13	344 ± 92*a,b*	*P* = 0.0003
8-Aminoinosine (n = 9)	69 ± 7	274 ± 80*a*	371 ± 142*a*	*P* = 0.0012
8-Aminohypoxanthine (n = 6)	94 ± 17	301 ± 69*a*	463 ± 40*a,b*	*P* < 0.0001

NS, not significant; n, sample size.

*^a^P* < 0.05 versus period 1 in Bonferroni test.

*^b^P* < 0.05 versus period 2 in Bonferroni test.

### Effects of 8-Aminopurines on Sodium Excretion

Administration of the vehicle for 8-aminopurines did not alter sodium excretion (Table 2). In contrast, 8-aminoguanine, 8-aminoinosine, and 8-aminohypoxanthine significantly (*P* < 0.0001, *P* = 0.0008, *P* < 0.0001, respectively) increased sodium excretion (Table 2). These results demonstrate that all three 8-aminopurines are natriuretics. However, during the third urine collection period (85–115 minutes into the protocol), the natriuretic response to 8-aminoinosine was less than the natriuretic response to 8-aminoguanine (sodium excretion during the third period for 8-aminoguanine, 8-aminoinosine, and 8-aminohypoxanthine was 67.2 ± 12.3, 24.2 ± 10.7, and 49.4 ± 7.9 µmoles per 30 minutes, respectively; one-factor ANOVA, *P* = 0.0342; Bonferroni test, *P* < 0.05 for 8-aminoguanine compared with 8-aminoinosine).

**TABLE 2:** Effects of 8-aminopurines on sodium excretion (all values are in units of µmoles per 30 min) All groups received their respective treatments just after period 1 (basal period). The 8-aminopurines were administered intravenously at 33.5 µmol/kg. *P* values are from repeated-measures one-factor analysis of variance. Values are means ± S.E.M.

Treatment Group	Period 1 (0–30 min)	Period 2 (40–70 min)	Period 3 (85–115 min)	*P* Value
Vehicle group (n = 6)	3.3 ± 1.0	4.4 ± 1.4	4.6 ± 1.8	NS
8-Aminoguanine (n = 5)	3.0 ± 0.9	11.0 ± 3.6	67.2 ± 12.3*a,b*	*P* < 0.0001
8-Aminoinosine (n = 9)	2.7 ± 0.5	12.8 ± 4.0*a*	24.2 ± 10.7*a*	*P* = 0.0008
8-Aminohypoxanthine (n = 6)	3.3 ± 0.6	23.0 ± 8.8*a*	49.4 ± 7.9*a,b*	*P* < 0.0001

NS, not significant; n, sample size.

*^a^P* < 0.05 versus period 1 in Bonferroni test

*^b^P* < 0.05 versus period 2 in Bonferroni test.

### Effects of 8-Aminopurines on Glucose Excretion

Administration of the vehicle for 8-aminopurines did not alter glucose excretion (Table 3). In contrast, 8-aminoguanine, 8-aminoinosine, and 8-aminohypoxanthine significantly (*P* < 0.0001, *P* = 0.0116, *P* = 0.0002, respectively) increased glucose excretion (Table 3). These results demonstrated that all three 8-aminopurines exert glucosuric effects. However, during the third urine collection period (85–115 minutes into the protocol), glucosuric responses were different in the three groups (8-aminoguanine > 8-aminohypoxanthine > 8-aminoinosine). Glucose excretion during the third period for 8-aminoguanine, 8-aminoinosine, and 8-aminohypoxanthine was 269 ± 55, 8.1 ± 2.0, and 28.6 ± 6.0 µg per 30 minutes, respectively (one-factor ANOVA, *P* < 0.0001; Bonferroni test, *P* < 0.05 for all comparisons among groups).

**TABLE 3:** Effects of 8-aminopurines on glucose excretion (all values are in units of µg per 30 min) All groups received their respective treatments just after period 1 (basal period). The 8-aminopurines were administered intravenously at 33.5 µmol/kg. *P* values are from repeated-measures one-factor analysis of variance. Values are means ± S.E.M.

Treatment Group	Period 1 (0–30 min)	Period 2 (40–70 min)	Period 3 (85–115 min)	*P* Value
Vehicle group (n = 6)	7.1 ± 2.4	7.6 ± 2.6	9.0 ± 2.4	NS
8-Aminoguanine (n = 5)	3.5 ± 0.9	35.7 ± 8.9*a*	269 ± 55*a,b*	*P* < 0.0001
8-Aminoinosine (n = 9)	3.6 ± 0.8	11.7 ± 3.9*^a^*	8.1 ± 2.0*a*	*P* = 0.0116
8-Aminohypoxanthine (n = 6)	6.1 ± 1.1	18.4 ± 4.5*^a^*	28.6 ± 6.0*a,b*	*P* = 0.0002

NS, not significant; n, sample size.

*^a^P* < 0.05 versus period 1 in Bonferroni test.

*^b^P* < 0.05 versus period 2 in Bonferroni test.

### Effects of 8-Aminopurines on Potassium Excretion

Administration of vehicle did not alter potassium excretion (Table 4). 8-Aminoguanine significantly decreased potassium excretion (*P* = 0.0003), whereas neither 8-aminoinosine nor 8-aminohypoxanthine affected potassium excretion (Table 4). These results demonstrated that 8-aminoinosine and 8-aminohypoxanthine differ in comparison with 8-aminoguanine in their overall electrolyte excretion profile.

**TABLE 4:** Effects of 8-aminopurines on potassium excretion (all values are in units of µmoles per 30 min) All groups received their respective treatments just after period 1 (basal period). The 8-aminopurines were administered intravenously at 33.5 µmol/kg. *P* values are from repeated-measures one-factor analysis of variance. Values are means ± S.E.M.

Treatment Group	Period 1 (0–30 min)	Period 2 (40–70 min)	Period 3 (85–115 min)	*P* Value
Vehicle group (n = 6)	34.5 ± 11.7	36.7 ± 8.8	51.1 ± 10.7	NS
8-Aminoguanine (n = 5)	21.3 ± 6.8	1.9 ± 0.4*a*	9.2 ± 2.4*b*	*P* = 0.0003
8-Aminoinosine (n = 9)	25.0 ± 4.6	24.8 ± 8.5	26.9 ± 9.0	NS
8-Aminohypoxanthine (n = 6)	24.9 ± 3.8	30.3 ± 14.8	30.0 ± 5.7	NS

NS, not significant; n, sample size.

*^a^P* < 0.05 versus period 1 in Bonferroni test.

*^b^P* < 0.05 versus period 2 in Bonferroni test.

### Effects of 8-Aminopurines on Hemodynamics

Table 5 summarizes the acute effects of vehicle (control), 8-aminoguanine, 8-aminoinosine, and 8-aminohypoxanthine on MABP, HR, RBF, and MBF. Vehicle, 8-aminoguanine, and 8-aminoinosine did not affect MABP, HR, RBF, or MBF. 8-Aminohypoxanthine did not affect MABP; however, 8-aminohypoxanthine caused a very slight, but statistically significant, decreased in HR and RBF. Also, 8-aminohypoxanthine briefly (period 2 only) increased MBF. These data indicate that the acute hemodynamic changes did not influence the renal excretory effects of these 8-aminopurines.

**TABLE 5:** Effects of 8-aminopurines on mean arterial blood pressure, heart rate, renal blood flow, and mesenteric blood flow All groups received their respective treatments just after period 1 (basal period). The 8-aminopurines were administered intravenously at 33.5 µmol/kg. *P* values are from repeated-measures one-factor analysis of variance. Values are means ± S.E.M.

	Period 1	Period 2	Period 3	*P* Value
	Mean Arterial Blood Pressure (mm Hg)			
Vehicle (n = 6)	112 ± 4	108 ± 3	107 ± 3	NS
8-Aminoguanine (n = 5)	102 ± 5	103 ± 4	103 ± 4	NS
8-Aminoinosine (n = 9)	108 ± 3	114 ± 4	109 ± 2	NS
8-Aminohypoxanthine (n = 6)	110 ± 3	120 ± 8	117 ± 6	NS
	Heart Rate (beats/min)			
Vehicle (n = 6)	404 ± 12	390 ± 16	384 ± 20	NS
8-Aminoguanine (n = 5)	377 ± 13	372 ± 14	372 ± 13	NS
8-Aminoinosine (n = 9)	398 ± 16	395 ± 14	389 ± 14	NS
8-Aminohypoxanthine (n = 6)	393 ± 7	380*^a^* ± 4	372*^a^* ± 6	0.0005
	Renal Blood Flow (ml/min)			
Vehicle (n = 6)	7.8 ± 1.2	7.8 ± 1.1	8.2 ± 1.0	NS
8-Aminoguanine (n = 5)	5.9 ± 0.7	5.2 ± 0.6	5.5 ± 0.8	NS
8-Aminoinosine (n = 9)	7.4 ± 0.5	6.6 ± 0.5	6.8 ± 0.4	NS
8-Aminohypoxanthine (n = 6)	8.2 ± 0.9	6.7*^a^* ± 0.5	6.8*^a^* ± 0.6	0.0244
	Mesenteric Blood Flow (ml/min)			
Vehicle (n = 6)	13.3 ± 2.4	13.0 ± 2.0	12.7 ± 1.8	NS
8-Aminoguanine (n = 5)	9.8 ± 2.2	10.8 ± 2.4	13.1 ± 2.6	NS
8-Aminoinosine (n = 9)	11.8 ± 1.0	11.9 ± 1.1	11.3 ± 1.1	NS
8-Aminohypoxanthine (n = 6)	14.5 ± 3.1	16.1*^b^* ± 3.6	12.8 ± 2.3	0.0400

NS, not significant; n, sample size.

*^a^P* < 0.05 versus period 1.

*^b^P* < 0.05 versus period 3.

### Effects of 8-Aminoinosine on 8-Aminohypoxanthine Excretion

**Fig. 5.**
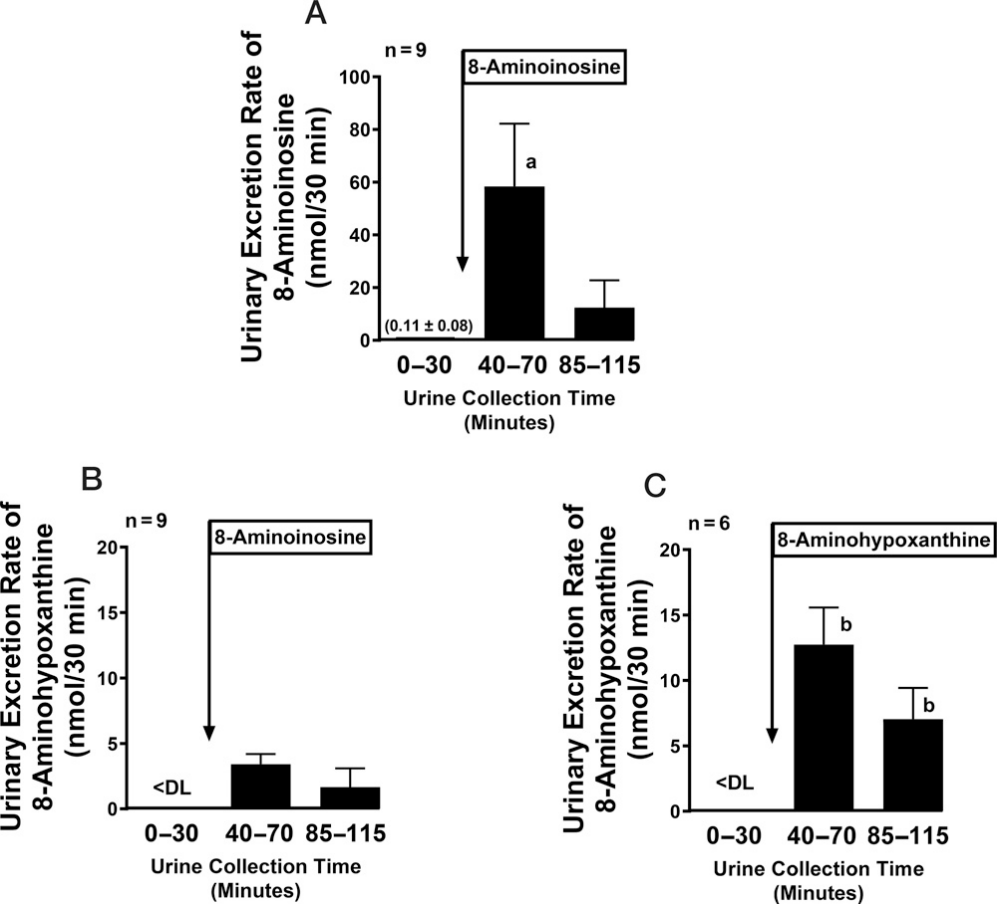
Effects of 8-aminoinosine and 8-aminohypoxanthine on the urinary excretion rate of 8-aminohypoxanthine. Bar graphs show effects of intravenous boluses of 8-aminoinosine (33.5 µmol/kg) on the urinary excretion rate (nmol per 30 minutes) of (A) 8-aminoinosine versus (B) 8-aminohypoxanthine. For comparison, also shown is the effect of intravenous boluses of 8-aminohypoxanthine (33.5 µmol/kg) on the urinary excretion rate (nmol per 30 minutes) of (C) 8-aminohypoxanthine. Values are means and S.E.M. for the indicated samples size (n). a, *P* < 0.05 versus same period in B; b, *P* < 0.05 versus same periods in B.

8-Aminoinosine is not only a competitive substrate for PNPase but is also metabolized to a potent competitive inhibitor, namely 8-aminohypoxanthine. To assess whether both of these mechanisms contribute to the renal effects of 8-aminoinosine, we measured the urinary excretion rate of 8-aminoinosine and 8-aminohypoxanthine following administration of 8-aminoinosine or 8-aminohypoxanthine. Administration of 8-aminoinosine increased the urinary excretion rate of both 8-aminoinosine (Fig. 5A) and 8-aminohypoxanthine (Fig. 5B); however, administration of 8-aminohypoxanthine increased the urinary excretion rate of 8-aminohypoxanthine significantly (*P* < 0.05) more so than did 8-aminoinosine (compare in Fig. 5, B and C). Also, administration of 8-aminoinosine increased the urinary excretion rate of 8-aminoinosine significantly (*P* < 0.05) more so than the urinary excretion rate of 8-aminohypoxanthine (compare in Fig. 5, A and B). These results suggest that although both mechanisms contribute to the renal effects of 8-aminoinosine, substrate competition is likely more important.

### Detection of 8-Aminoinosine in Urine

**Fig. 6.**
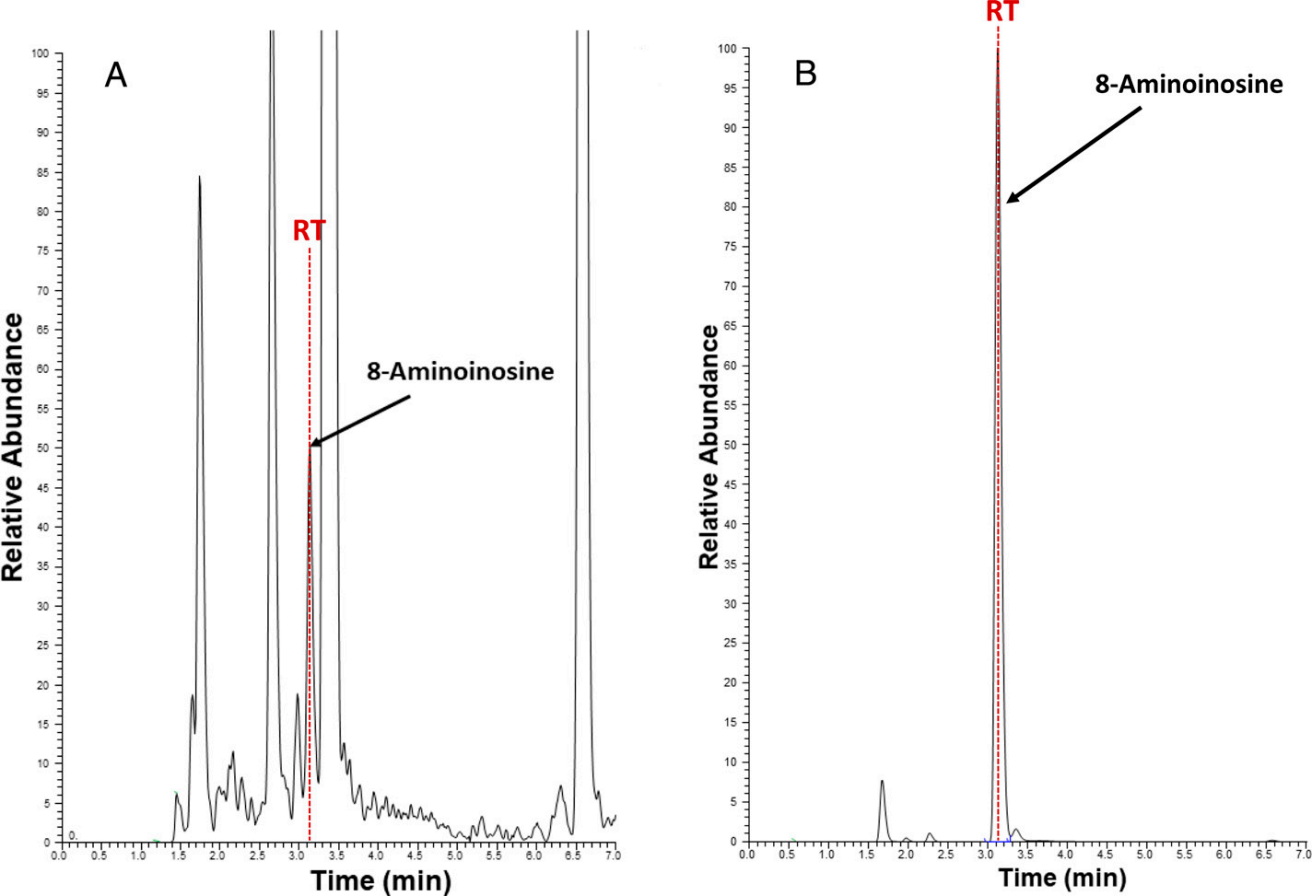
Detection of 8-aminoinosine in urine. Illustration of chromatograms from UPLC-MS/MS analysis of urine (A) before and (B) after administration of 8-aminoinosine (33.5 µmol/kg). The monitored transition was: 284 m/z (precursor ion) → 152 m/z (product ion). As shown, even in the sample before injection of 8-aminoinosine, there was a peak that matched the transition and retention time (RT) observed for the large peak of 8-aminoinosine after injection.

Fig. 6 illustrates chromatograms from the UPLC-MS/MS analysis of urine before (Fig. 6A) and after (Fig. 6B) administration of 8-aminoinosine (33.5 µmol/kg). The monitored mass transition (precursor ion of 284 m/z and product ion of 152 m/z) was selected to detect 8-aminoinosine. As shown, even in the sample before administration of 8-aminoinosine, there was a peak that precisely matched the mass transition and retention time observed for the large peak of 8-aminoinosine after administration of 8-aminoinosine. This result indicates that 8-aminoinosine is an endogenous 8-aminopurine.

### Effects of 8-Aminohypoxanthine on Xanthine Oxidase Activity

Xanthine oxidase metabolizes hypoxanthine to xanthine. Because 8-aminohypoxanthine is a hypoxanthine analog, it is possible that 8-aminohypoxanthine inhibits xanthine oxidase. To test this, we incubated xanthine oxidase (0.2, 0.4, 1, or 2 µg; incubation time, 15 minutes) with hypoxanthine (200 µmol/L) and with or without 8-aminohypoxanthine (200 µmol/L). After the incubation, hypoxanthine, xanthine, uric acid, 8-aminohypoxanthine, and 8-aminoxanthine were quantified by HPLC using area under the chromatographic peak following equal-volume injections of the reaction mixtures. The results of these experiments are summarized in Table 6. With low amounts of xanthine oxidase (0.2 µg), hypoxanthine levels decreased, and xanthine and uric acid levels increased. As the amount of xanthine oxidase was further increased to 0.4, 1, and 2 µg, the peak areas due to hypoxanthine and xanthine decreased, becoming undetectable with 1 and 2 µg of xanthine oxidase, whereas levels of uric acid increased. These results were not affected by coincubation of the xanthine oxidase/hypoxanthine mixture with 8-aminohypoxanthine, thus indicating that 8-aminohypoxanthine does not inhibit xanthine oxidase. Notably, with increasing amounts of xanthine oxidase, 8-aminohypoxanthine levels decreased, whereas 8-aminoxanthine levels increased. This suggested that 8-aminohypoxanthine may be a substrate for xanthine oxidase.

**TABLE 6:** Lack of effects of 8-aminohypoxanthine (8-AminoHX) on xanthine oxidase Increasing amounts of xanthine oxidase were incubated for 15 minutes at 30°C in 50 mmol/L of KH_2_PO_4_ with substrate (hypoxanthine) and the potential inhibitor (8-aminohypoxanthine). The remaining hypoxanthine and 8-aminohypoxanthine (8-aminoHX) and generated products (xanthine, uric acid, and 8-aminoxanthine) were measured by HPLC and quantified as area under the chromatographic peak following equal volume injections (4 µl) of the reaction mixture.

Reaction Components			HPLC Results				
Xanthine Oxidase (µg)	[Hypoxanthine] (µmol/L)	[8-AminoHX] (µmol/L)	Hypoxanthine (Peak Area)	Xanthine (Peak Area)	Uric Acid (Peak Area)	8-AminoHX (Peak Area)	8-Aminoxanthine (Peak Area)
0	200	0	277	ND	ND	ND	ND
0.2	200	0	184	48	153	ND	ND
0.2	200	200	178	49	154	1979	ND
0.4	200	0	92	24	271	ND	ND
0.4	200	200	64	19	297	1051	20
1	200	0	ND	ND	406	ND	ND
1	200	200	ND	ND	399	796	68
2	200	0	ND	ND	437	ND	ND
2	200	200	ND	ND	404	586	108

ND, not detected.

### Kinetics of Xanthine Oxidase Metabolism of Hypoxanthine versus 8-Aminohypoxanthine

**Fig. 7.**
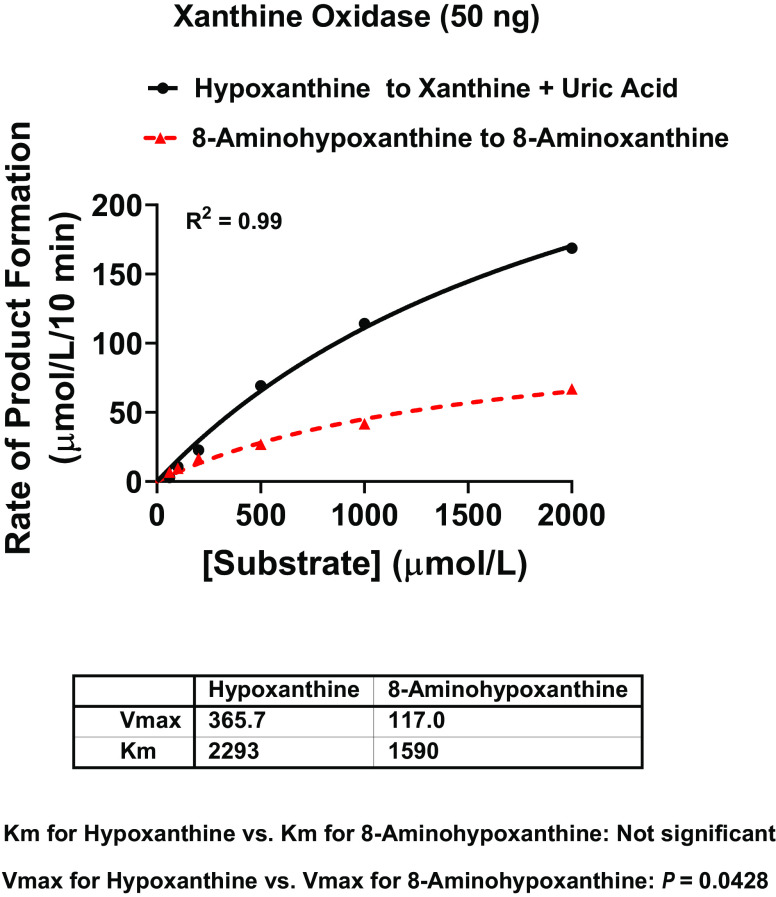
Kinetics of xanthine oxidase metabolism of hypoxanthine versus 8-aminohypoxanthine. Xanthine oxidase (50 ng) was incubated for 10 minutes at 30°C in 50 µl of medium containing 60–2000 µmol/L of hypoxanthine or 8-aminohypoxanthine, 0.1 mg/ml of bovine serum albumin, and 50 µmol/L of KH_2_PO_4_ (pH 7.4). Xanthine, uric acid, and 8-aminoxanthine were analyzed by HPLC with UV absorbance, and the rate of product formation was determined (xanthine plus uric acid for hypoxanthine as substrate and 8-aminoxanthine for 8-aminohypoxanthine as substrate). To determine Km (μmol/ L) and Vmax (μmol/L/10 min), results were fitted to the Michaelis-Menten equation using GraphPad Prism 9 for Windows (San Diego, CA). The Km of xanthine oxidase with 8-aminohypoxanthine as substrate was similar to that with hypoxanthine as substrate. The Vmax of xanthine oxidase with 8-aminohypoxanthine as substrate was approximately 32% of that with hypoxanthine as substrate.

To test further whether 8-aminohypoxanthine is a substrate for xanthine oxidase and to quantify how good a substrate 8-aminohypoxanthine is for xanthine oxidase, we performed enzyme kinetics on xanthine oxidase with 8-aminohypoxanthine versus hypoxanthine as substrates. Xanthine oxidase was incubated for 10 minutes with 60–2000 µmol/L of 8-aminohypoxanthine or hypoxanthine; xanthine, uric acid, and 8-aminoxanthine were analyzed by HPLC with UV absorbance; and the rate of product formation was determined (xanthine plus uric acid for hypoxanthine as substrate and 8-aminoxanthine for 8-aminohypoxanthine as substrate). To determine Km and Vmax, results were fitted to the Michaelis-Menten equation using GraphPad Prism 9 for Windows (San Diego, CA). The Km of xanthine oxidase with 8-aminohypoxanthine as substrate was similar to, and not statistically different from, the Km of xanthine oxidase with hypoxanthine as substrate (Fig. 7**)**. The Vmax of xanthine oxidase with 8-aminohypoxanthine as substrate was approximately 32% of the Vmax with hypoxanthine as substrate. Thus, although xanthine oxidase processes hypoxanthine more efficiently than 8-aminohypoxanthine, these results confirm that 8-aminohypoxanthine is a good substrate for xanthine oxidase.

## Discussion

As previously reported by us, 8-aminoguanine and 8-aminoguanosine have a unique pharmacological profile in that they induce diuresis, natriuresis, glucosuria, and antikaliuresis (Jackson et al., 2016). Our subsequent studies showed that the renal excretory effects of 8-aminoguanosine, with the exception of antikaliuresis, are mediated by metabolism of 8-aminoguanosine to 8-aminoguanine (Jackson and Mi, 2017). With regard to mechanism, the diuretic, natriuretic, and glucosuric effects of 8-aminoguanine and 8-aminoguanosine (via conversion to 8-aminoguanine) are due to inhibition of PNPase (Jackson et al., 2018). On the other hand, antikaliuresis is likely mediated by a distinct mechanism, possibly involving in part inhibition of Rac1 signaling (Jackson et al., 2018), although this hypothesis requires further evaluation. How inhibition of PNPase induces diuresis, natriuresis, and glucosuria is under investigation.

**Fig. 8.**
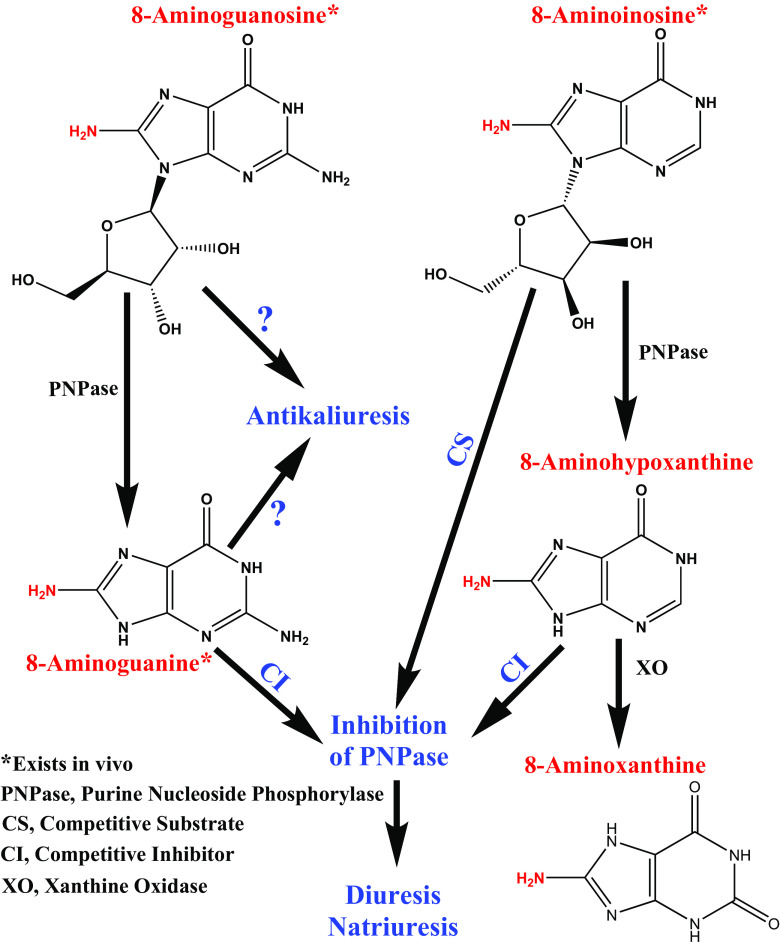
Model for renal pharmacology of 8-aminopurines. This illustration summarizes our hypothesis for how 8-aminopurines affect renal excretory function and is based on our previous studies and the present findings. We propose that 8-aminoguanosine and 8-aminoguanine have direct effects to suppress potassium excretion independent of PNPase inhibition and possibly in part via inhibition of Rac1 signaling. Neither 8-aminoinosine nor 8-aminohypoxanthine alter potassium excretion. 8-Aminoguanine potently inhibits PNPase, which in turn causes diuresis, natriuresiss, and glucosuria. Most of the effects of 8-aminoguanosine on urine volume and sodium and glucose excretion are mediated via its conversion to 8-aminoguanine. 8-Aminohypoxanthine and 8-aminoinosine, via inhibition of PNPase, also cause diuresis, natriuresis, and modest glucosuria. 8-Aminoinosine directly reduces PNPase activity via substrate competition and also indirectly blocks PNPase via its conversion to 8-aminohypoxanthine. 8-Aminoguanosine, 8-aminoguanine, and 8-aminoinosine are endogenous 8-aminopurines. Possibly, 8-aminohypoxanthine is also endogenous but is so rapidly metabolized to 8-aminoxanthine that it is difficult to detect. Whether 8-aminoxanthine exists in vivo and has biologic activity is currently unknown. 8AG, 8-aminoguanine; 8AHX, 8-aminohypoxanthine; 8AI, 8-aminoinosine; CI, competitive inhibitor; CS, competitive substrate; PNPase, purine nucleoside phosphorylase; XO, xanthine oxidase.

Because 8-aminoguanosine and 8-aminoguanine are 8-aminopurines, we were motivated to determine whether other 8-aminopurines may have effects on the kidneys, similar to those elicited by 8-aminoguanosine and 8-aminoguanine. In this regard, 8-aminoinosine is similar in structure to 8-aminoguanosine, and 8-aminohypoxanthine is similar in structure to 8-aminoguanine (Fig. 8). Indeed, 8-aminoinosine and 8-aminohypoxanthine differ from 8-aminoguanosine and 8-aminoguanine, respectively, only in the absence of an amino group in the 2 position of the purine ring (Fig. 8).

As mentioned, with the exception of antikaliuresis, the effects of 8-aminoguanoisne and 8-aminohypoxanthine on renal excretory function are mediated by inhibition of PNPase (Jackson et al., 2018). Therefore, we began our examination of 8-aminoinosine and 8-aminohypoxathine by comparing their potencies as PNPase inhibitors to that of 8-aminoguanine. These enzyme kinetic experiments showed that, similar to 8-aminoguanine, both 8-aminoinosine and 8-aminohypoxanthine are PNPase inhibitors. In this regard, 8-aminoinosine and 8-aminohypoxanthine are approximately 10-fold less potent than 8-aminoguanine, and the potency of 8-aminoinosine is similar to the potency of 8-aminohypoxanthine. These findings suggested that both 8-aminoinosine and 8-aminohypoxanthine should have qualitative effects on urine volume and sodium and glucose excretion, similar to those of 8-aminoguanine. However, it would be expected that at similar doses, the renal excretory effects of both compounds would be quantitatively less than those produced by 8-aminoguanine.

Independent of our studies, Rabuffetti et al. (2021) also observed that 8-aminoinosine is an inhibitor of PNPase, and their kinetic analysis suggested that 8-aminoinosine is a competitive inhibitor of this enzyme. Our results indicate that 8-aminoinosine’s ability to block PNPase activity likely involves two mechanisms. Here, we show that 8-aminoinosine is an excellent substrate for rhPNPase and is readily converted to the product 8-aminohypoxanthine, with a Km and Vmax similar to that of PNPase’s natural substrate inosine. Indeed, inosine competitively prevents the metabolism of 8-aminoinosine to 8-aminohypoxanthine. These findings suggest that 8-aminoinosine is more precisely characterized as a competitive substrate, rather than a competitive inhibitor, of PNPase. In addition to being a competitive substrate, metabolism of 8-aminoinosine also generates a potent competitive inhibitor of PNPase, namely 8-aminohypoxanthine. Therefore, it can be concluded that 8-aminoinosine blocks PNPase activity by two mechanisms: 1) 8-aminoinosine is a competitive substrate for PNPase, and 2) 8-aminoinosine is converted to the potent PNPase inhibitor 8-aminohypoxanthine.

Consistent with our in vitro analysis of the effects of 8-aminoguanosine, 8-aminoinosine, and 8-aminohypoxanthine on rhPNPase, we observed that administration of these compounds to rats suppressed both the guanine-to-guanosine ratio and the hypoxanthine-to-inosine ratio in urine. This is as expected for inhibitors of an enzyme that converts guanosine to guanine and inosine to hypoxanthine. Also consistent with our in vitro kinetic analyses, the effects of 8-aminoguanine on these ratios were significantly greater than the effects of either 8-aminoinosine or 8-aminohypoxanthine.

In general, the effects of 8-aminoinosine and 8-aminohypoxanthine on urine volume and sodium excretion were similar to those of 8-aminoguanine, with the exception that the natriuretic effect of 8-aminoinosine was statistically less than the natriuretic effect of 8-aminoguanine. In contrast, the glucosuric effects of 8-aminoinosine and 8-aminohypoxanthine diverged from those of 8-aminoguanine. Although all three compounds increased glucose excretion, 8-aminoguanine was clearly superior in this regard. Possibly, this is because the glucosuric effects are mediated by PNPase inhibition (Jackson et al., 2018), and 8-aminoguanine more potently inhibits this enzyme. We hypothesize that the effects of PNPase inhibition on urine and sodium excretion are manifest at a lower threshold of PNPase inhibition than the effects of PNPase inhibition on glucose excretion. This could explain the relatively similar effects of the three compounds on urine volume and sodium excretion and the greater effects of 8-aminoguanine on glucose excretion. However, it is also possible that 8-aminoinosine and 8-aminohypoxanthine exert an additional diuretic/natriuretic mechanism that contributes to the efficacy of these two compounds or that the glucosuric effects of 8-aminoguanine entail an additional mechanism.

Notably, 8-aminoguanine elicits an antikaliuretic effect, whereas 8-aminohypoxanthine and 8-aminoxanthine do not. Previously, we showed that similar levels of PNPase inhibition using 8-aminoguanine versus 9-deazaguanine induced nearly identical increases in urine volume and sodium and glucose excretion (Jackson et al., 2018). In contrast, 9-deazaguanine had no effect on potassium excretion (Jackson et al., 2018). Instead, the Rac1 inhibitor NSC23766 mimicked the antikaliuretic effects of 8-aminoguanine (Jackson et al., 2018), and in vitro, we observed that both 8-aminoguanosine and 8-aminoguanine modestly reduced Rac1 activation (Jackson et al., 2018). Rac1 has been shown to directly activate mineralocorticoid receptors (Shibata et al., 2008; Shibata et al., 2011; Hirohama et al., 2021), a process that would increase potassium excretion. Our hypothesis is that 8-aminoguanosine and 8-aminoguanine induce antikaliuresis by a mechanism independent of PNPase inhibition and possibly in part due to inhibition of Rac1 signaling; however, because the effects of 8-aminoguanosine and 8-aminoguanine on activated Rac1 are modest, inhibition of Rac1 may not be the main mechanism by which 8-aminoguanosine and 8-aminoguanine affect potassium excretion. We tested in cultured mouse collecting duct cells whether 8-aminoinosine or 8-aminohypoxanthine inhibited Rac1 activation or the Rac1/Pak1 signaling pathway (data not shown) and found no evidence that either of these 8-aminopurines interferes with activation of Rac1 or downstream signaling engaged by Rac1. This may partially explain the inability of 8-aminoinosine and 8-aminohypoxanthine to induce antikaliuresis.

The present study, combined with our previously published results (Jackson et al., 2016; Jackson and Mi, 2017; Jackson et al., 2018), show that five different PNPase inhibitors (8-aminoguanosine, 8-aminoguanine, 8-aminoinosine, 8-aminohypoxanthine, and 9-deazaguanine) cause diuresis, natriuresis, and glucosuria. These results suggest that kidney PNPase importantly regulates renal excretory function; therefore, the distribution of PNPase in the kidney may be critically important. Currently, there are few published studies that address the regional distribution of PNPase in the kidney. Rubio and Berne (1980) reported that in the kidney, PNPase expression was greatest in the vasculature, particular in the cytoplasm of capillary endothelial cells. This finding suggests the possibility that PNPase in the renal microcirculation regulates renal excretory function, perhaps by influencing medullary blood flow. Takahashi et al. (1989) examined the relative activity of PNPase in glomeruli, proximal tubules (S1, S2, and S3 segments), cortical thick ascending limbs, distal convoluted tubules (including connecting tubules), and cortical collecting ducts. These investigators reported that all of these nephron regions express PNPase activity, with the greatest activity in glomeruli and substantial activity in cortical thick ascending limbs, distal convoluted tubules, and cortical collecting ducts. Even proximal tubule subsegments expressed PNPase activity, although less than other nephron regions. Together, these findings suggest that PNPase inhibition may influence renal excretory function by inhibiting PNPase at multiple nephron sites and the surrounding microcirculation.

Because hypoxanthine is a substrate for xanthine oxidase, we considered the possibility that 8-aminohypoxanthine is a competitive inhibitor of xanthine oxidase. Since xanthine oxidase is well known to generate reactive oxygen species (Bortolotti et al., 2021), if 8-aminohypoxanthine were to inhibit this reactive oxygen species–generating enzyme, this could be an important aspect of the pharmacology of 8-aminohypoxanthine. However, our initial efforts to evaluate this concept showed that 8-aminohypoxanthine does not interfere with the ability of xanthine oxidase to metabolize hypoxanthine to xanthine or xanthine to uric acid. Moreover, we observed that in the presence of xanthine oxidase, 8-aminohypoxanthine disappeared, and 8-aminoxanthine was detected instead. This led us to compare the kinetics of the conversion by xanthine oxidase of 8-aminohypoxanthine to 8-aminoxanthine versus hypoxanthine to xanthine. This analysis confirmed that 8-aminohypoxanthine is a good xanthine oxidase substrate.

8-Aminoguanosine and 8-aminoguanine are endogenous 8-aminopurines. Another important observation of the present study is that we detected in urine endogenous 8-aminoinosine. This indicates that there exist at least three different endogenous 8-aminopurines that are biologically active. Although we did not detect 8-aminohypoxanthine, this could be because this 8-aminopurine is rapidly metabolized to 8-aminoxanthine. Currently, we do not have a UPLC-MS/MS assay for 8-aminoxanthine, so we were unable to assess the in vivo existence of this compound. In future studies, we would like to investigate whether in vivo 8-aminohypoxanthine is converted to 8-aminoxanthine, whether both compounds exist in vivo, and whether 8-aminoxanthine has pharmacological activity. As mentioned, both 8-aminoguanosine and 8-aminoguanine attenuate deoxycorticosterone/salt hypertension (Jackson et al., 2016), age-associated pathology of the lower urinary tract (Birder et al., 2020), pulmonary hypertension (Jackson and Tofovic, 2020), and sickling of red blood cells in blood from patients living with sickle cell anemia (Jackson and Tofovic, 2020). It would also be important to investigate the relative efficacy of 8-aminoinosine and 8-aminohypoxanthine in these disorders.

The findings from our previous investigations (Jackson et al., 2016; Jackson and Mi, 2017; Jackson et al., 2018) along with the current findings are summarize in Fig. 8. We propose that 8-aminoguanosine and 8-aminoguanine have direct effects to suppress potassium excretion independent of PNPase inhibition and possibly in part via inhibition of Rac1 signaling (although this conclusion requires further investigation). Neither 8-aminoinosine nor 8-aminohypoxanthine alter potassium excretion. 8-Aminoguanine potently inhibits PNPase, which in turn causes diuresis, natriuresis, and glucosuria. Most of the effects of 8-aminoguanosine on urine volume and sodium and glucose excretion are mediated via its conversion to 8-aminoguanine. 8-Aminohypoxanthine and 8-aminoinosine, via inhibition of PNPase, also cause diuresis, natriuresis, and modest glucosuria. 8-Aminoinosine directly reduces PNPase activity via substrate competition and also indirectly blocks PNPase via its conversion to 8-aminohypoxanthine. 8-Aminoguanosine, 8-aminoguanine, and 8-aminoinosine are endogenous 8-aminopurines. Possibly, 8-aminohypoxanthine is also endogenous but is so rapidly metabolized to 8-aminoxanthine that it is difficult to detect. Whether 8-aminoxanthine exists in vivo and has biologic activity is currently unknown.

A caveat of the present study is the use of Inactin anesthesia. Anesthesia was necessary because rats were extensively instrumented to monitor not only blood pressure and heart rate but also renal blood flow, mesenteric blood flow, and urine output while giving intravenous infusions. Inactin anesthesia was selected because this anesthetic has been extensively used by the renal physiology community, with results that are consistent with studies in conscious animals. In the present study, we did not include a positive control, i.e., a standard diuretic. This is because we previously reported the results of a head-to-head comparison of 8-aminoguanine with amiloride at equivalent doses (Jackson et al., 2016). We found that the effects of 8-aminoguanine on sodium excretion were greater than the effects of amiloride.

In conclusion, 8-aminopurines have potentially useful pharmacological profiles. For diuresis and natriuresis accompanied by glucosuria and antikaliuresis, 8-aminoguanine (or its prodrug 8-aminoguanosine) would be preferred. If only diuresis and natriuresis, without marked glucosuria or antikaliuresis, is desired, 8-aminohypoxanthine or 8-aminoinosine might be useful. Finally, here we report the in vivo existence of another pharmacologically active 8-aminopurine, namely 8-aminoinosine.

## Abbreviations

HPLC: high pressure liquid chromatography
HR: heart rate
Ki: inhibition constant
Km: Michaelis constant
MABP: mean arterial blood pressure
MBF: mesenteric blood flow
PE: polyethylene
PNPase: purine nucleoside phosphorylase
RBF: renal blood flow
rhPNPase: recombinant human purine nucleoside phosphorylase
UPLC-MS/MS: ultraperformance liquid chromatography–tandem mass spectrometry
Vmax: maximum reaction velocity
